# Propulsion of magnetically actuated achiral planar microswimmers in Newtonian and non-Newtonian fluids

**DOI:** 10.1038/s41598-021-00153-5

**Published:** 2021-10-27

**Authors:** Zhi Chen, Zihan Wang, David Quashie, Prateek Benhal, Jamel Ali, Min Jun Kim, U Kei Cheang

**Affiliations:** 1 Department of Mechanical and Energy Engineering, Southern University of Science and Technology, Shenzhen, Guangdong China; 2 Department of Chemical and Biomedical Engineering, FAMU-FSU College of Engineering, Tallahassee, FL USA; 3 National High Magnetic Field Laboratory, Tallahassee, FL USA; 4 Department of Mechanical Engineering, Southern Methodist University, Dallas, TX USA

**Keywords:** Biomedical engineering, Applied physics, Fluid dynamics

## Abstract

Magnetic achiral planar microswimmers can be massively fabricated at low cost and are envisioned to be useful for in vivo biomedical applications. To understand locomotion in representative in vivo environments, we investigated the swimming performance of achiral planar microswimmers in methylcellulose solutions. We observed that these microswimmers displayed very similar swimming characteristics in methylcellulose solutions as in water. Furthermore, this study indicated that the range of precession angles increased as the concentration of MC solution increased. Last, it was demonstrated that achiral planar microswimmers with similar precession angles exhibited nearly the same dimensionless speeds in different concentrations of the methylcellulose solutions. Upon understanding swimmer kinematics, more effective control over the achiral planar microswimmers can be achieved to perform multiple biomedical tasks in in vivo environments.

## Introduction

Magnetic microswimmers used for in vivo biomedical applications, such as drug delivery, minimally invasive surgery, and tissue engineering^1,2^, will encounter non-Newtonian biofluids^3^. While achiral planar microswimmers, which are 2D planar achiral-shaped structures capable of swimming in bulk fluid upon actuation by a rotating magnetic field, in Newtonian fluids have been studied extensively^4–6^, there are no reported studies on achiral planar microswimmers in non-Newtonian fluids. Past studies of microswimmers in non-Newtonian fluids focused on spiral-type microswimmers. Berg et al. investigated the locomotion behavior of *Leptospira* in a high-viscosity Methyl cellulose (MC) solution and indicated that the enhanced propulsion efficiency was because the thin flagella of the bacteria can pass through the viscoelastic polymer network and produce corkscrew motion by interacting with the network^7^. Peyer et al. studied the swimming performance of artificial bacterial flagella in different MC solutions and found that a higher concentration of MC solution was conducive to its swimming propulsion^8^. Schamel et al. placed spiral nanoswimmers in hyaluronic acid and revealed that their swimming speed in hyaluronic acid was faster than that in a Newtonian fluid^9^. These representative studies concluded that non-Newtonian fluids do not hinder movement, but enhances the propulsion efficiency of spiral micro/nanoswimmers; thus, demonstrating their potential for in vivo locomotion. To validate the feasibly of achiral planar micro/nanoswimmers on in vivo applications, it is critical to study whether non-Newtonian fluids will hinder or enhance achiral propulsion microswimmers. The properties of the MC solutions can be tuned by changing the concentration (Newtonian or non-Newtonian fluid). Although the MC solutions are not a replacement for biological fluids which these swimmers might encounter in vivo, they can provide tunable parameters to study the swimming performance of achiral microswimmers in fibrous environments, which can give some insights into the complex biological fluids they might encounter in vivo.

Various propulsion methods for microswimmers at low Reynolds number were developed, such as spiral propulsion of bacterial flagella^10^, traveling wave propulsion of sperm cell^11^, and catalytic propulsion of chemically driven microswimmers^12^. Although these microswimmers exhibit excellent mobility, the fabrication process remained rather complex, costly, and/or yielded low throughput. The three-beads microswimmers demonstrated that achiral microswimmers could acquire chirality and achieve forward motion under rotating magnetic field^13^. Moreover, their swimming ability and hydrodynamics have been demonstrated through experiments^6,14^. It was theoretically demonstrated that achiral planar shapes are nearly optimal propellers^15^. Furthermore, the control of multiple achiral microswimmers is possible by exploiting the discrepancy in magnetic moment^5^. Through low-cost and high-throughput photolithography technology, achiral planar microswimmers can be manufactured in large batches with high consistency. Mu et al*.* have successfully realized targeted drug delivery photolithography manufactured MOFs-based micro/nanorobots, which demonstrated the potential to use achiral planar microswimmers for biomedical applications^16^. In order to realize the use of achiral planar microswimmers for in vivo applications, it is important to explore their swimming performance in biological-like fluids*.* In short, achiral planar microswimmers are a promising candidate in the field of micro/nanorobotics. To demonstrate their feasibility for in vivo applications, there is an urgency to develop an understanding of the swimming capability of achiral planar microswimmers in non-Newtonian fluids. Specifically, although the MC solutions are not fully representative of the in vivo biological fluids, this study systematically examines swimming in methylcellulose solutions in order to gain insights into the locomotion of achiral planar microrobots in the fibrous network of the extracellular matrix.

## Swimming in viscous environments

**Figure 1:**
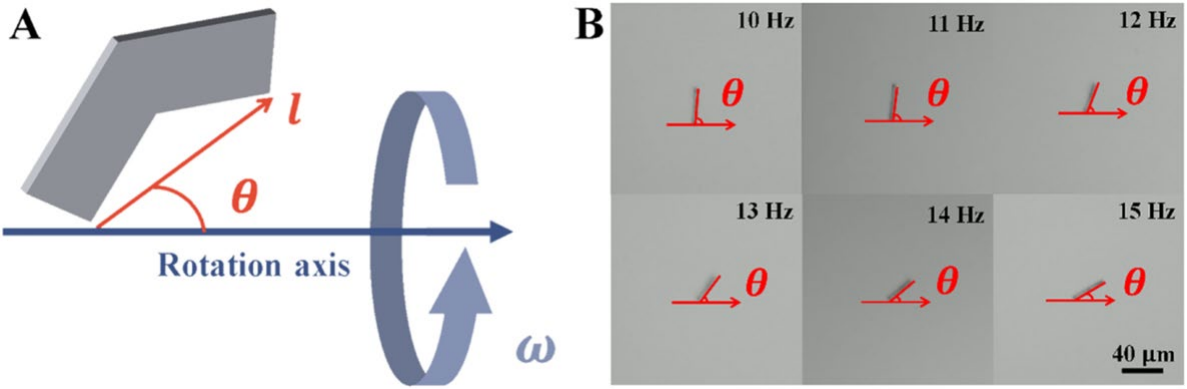
(**A**) Schematic of achiral planar microswimmer’s precession angle under rotation magnetic field; (**B**) Precession angles at different frequencies (10–15 Hz).

In this article, the motion of achiral planar microswimmers was examined in different concentrations of MC solution (0.2% w/v, 0.4% w/v, and 0.6% w/v). The effects of MC concentration on propulsion were quantitatively analyzed by observing the microswimmers’ swimming velocities and precession angles. Precession angle is defined by the angle $\theta$ between the rotation axis and the easy axis $l$ of the microswimmer, as shown in Fig. 1A. It was found that the precession angle with increasing rotational frequency under constant magnetic field strength. However, if the precession angle remains the same, the swimming speed of the achiral microswimmers in any fluid, Newtonian or not, will be very similar. Therefore, achiral planar microswimmers can swim in non-Newtonian fluids without hindrance by managing the change in precession angle, as was done in water^6,14^. We carried out effective motion control by maintaining similar precession angles in water and the MC solutions, as shown in the Fig S3. This is demonstrative of how controlling the precession angle of the microswimmers can lead to similar swimming performance of the swimmers in different fluids; thus, demonstrates effective motion control.

In stokes fluid flows, the mobility model of achiral planar microswimmers can be expressed by^4,17^.

1 $$\left(\begin{array}{c}{\varvec{V}}\\ \boldsymbol{\Omega }\end{array}\right)=\left(\begin{array}{cc}{\varvec{K}}& {\varvec{C}}\\ {{\varvec{C}}}^{{\varvec{T}}}& {\varvec{M}}\end{array}\right)\left(\begin{array}{c}{\varvec{F}}\\ {\varvec{N}}\end{array}\right)$$

where ***K***, ***M***, and ***C*** are submatrices that relate the translational velocity (***V***) and force (***F***), rotational velocity (***Ω***) and torque (***N***), and translational velocity and torque, respectively. As achiral planar microswimmers are driven by the magnetic torque, ${\varvec{F}}=0$ can be assumed which yields:

2 $$\boldsymbol{\Omega }={\varvec{M}}\cdot {\varvec{N}}$$

3 $${\varvec{V}}={\varvec{C}}\cdot {\varvec{N}}$$

Combining Eqs. (2) and (3) to obtain

4 $${\varvec{V}}={\varvec{C}}\cdot {{\varvec{M}}}^{-1}\cdot \boldsymbol{\Omega }$$

The variation of the precession angle will result in changes to submatrices ${\varvec{M}}$ and ${\varvec{C}}$, which explains the non-linear relationship between achiral planar microswimmers’ velocity and rotating frequency. To maintain steady rotation, the precession angle of achiral planar microswimmers will change with the frequency of the rotating magnetic field, thus resulting in the non-linear velocity-frequency curve.

**Figure 2:**
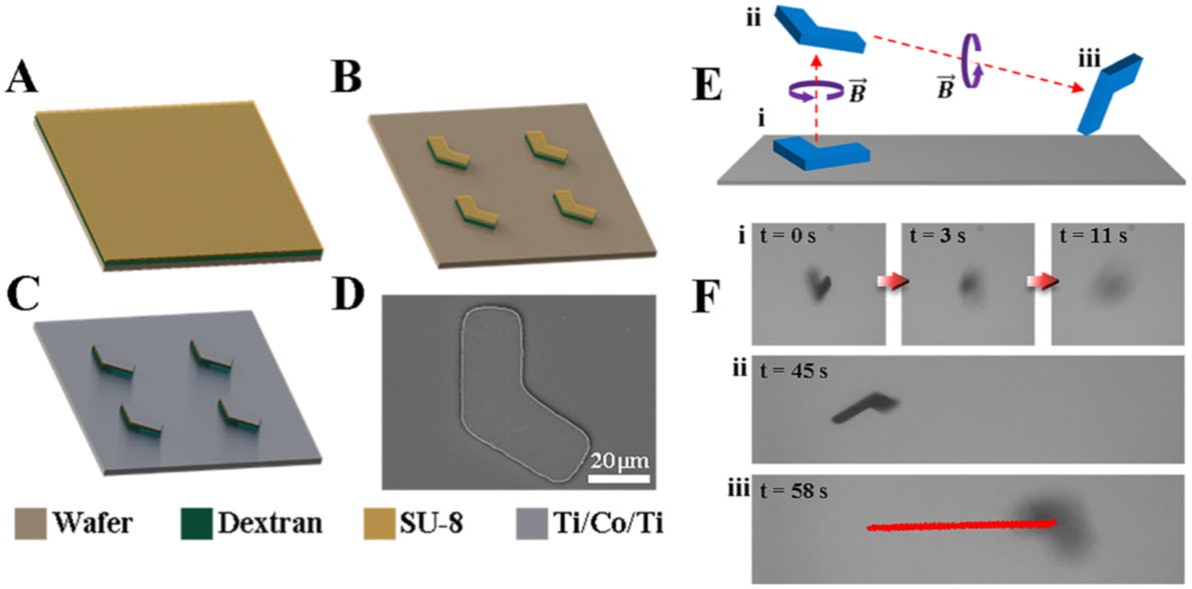
(**A**–**C**) Fabrication process of achiral microswimmers. (**D**) Scanning electron microscope (SEM) image of an achiral microswimmer. The scale bar is 20 μm. (**E**,**F**) 3D swimming of achiral planar microswimmers. (**E**) Schematic and (**F**) experiment of swimming in 3D space. (Multimedia view from video S1).

The fabrication procedure of achiral planar microswimmers using conventional photolithography and electron beam evaporation is described in the methods, as shown in Fig. 2A–C. Photolithography allowed for the fabrication of achiral planar SU-8 microstructures. Next, electron beam evaporation was used to deposit titanium-cobalt-titanium nano-layers on the SU-8 structures, which endowed the microswimmers with magnetic properties and biocompatibility. An SEM image of a representative microswimmer is shown in Fig. 2D. Afterward, we measured the direction of magnetic moment, shown in Fig S4. The microswimmers are demonstrated swimming in 3D space in an experiment shown in Fig. 2E,F where a representative achiral planar microswimmer (i) started on the substrate, (ii) swam in the z-direction away from the substrate, and (iii) then swam in the x-direction in bulk fluid while slowly sinking.

## Results

**Figure 3:**
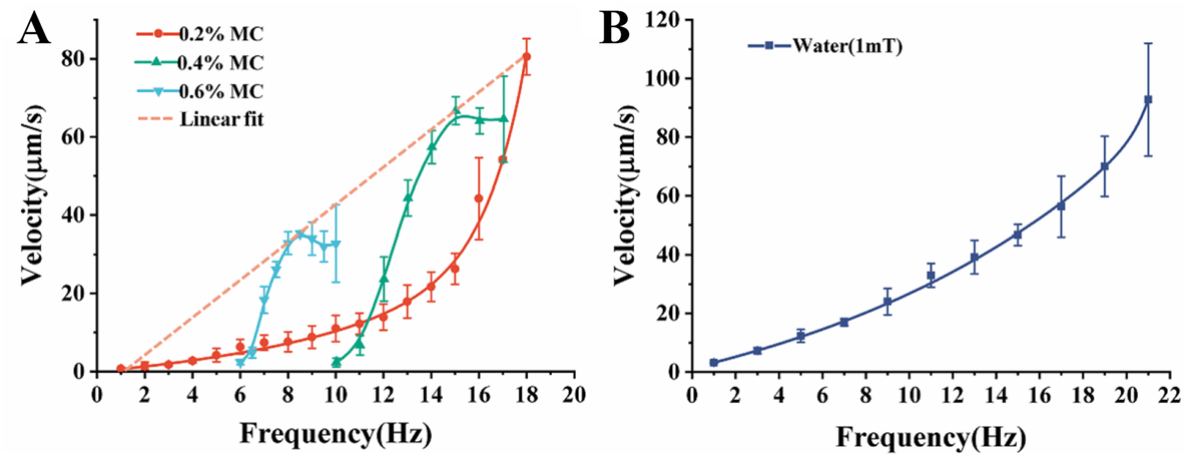
Velocity measurements in MC solutions and water. (**A**) Velocity curves for 0.6%, 0.4%, and 0.2% MC and a linear fit on peak average velocities. (**B**) Velocity curve for water. The error represents standard error with a sample size of three.

**Figure 4:**
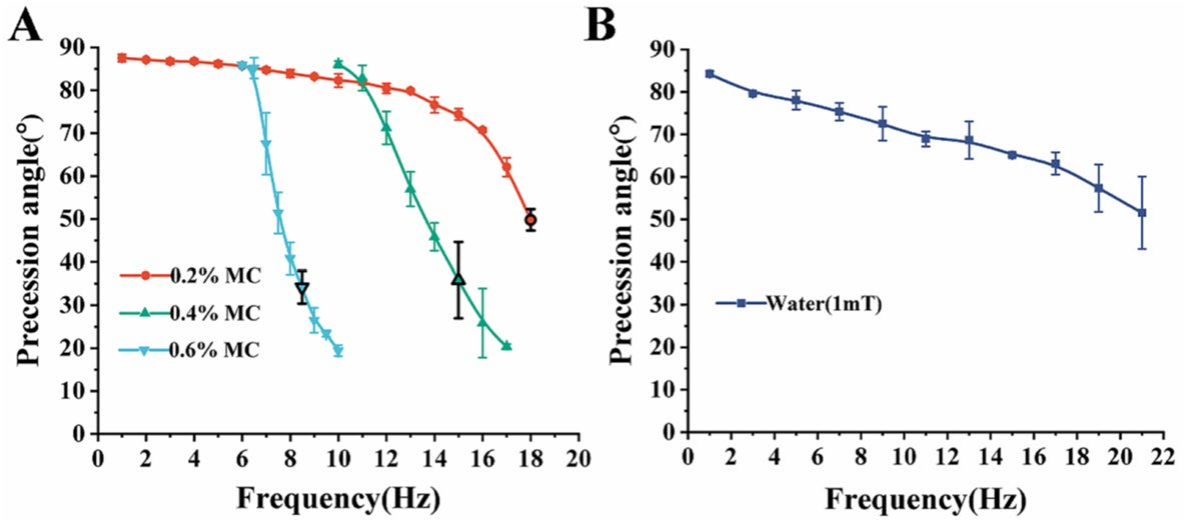
Precession angle measurements in MC solutions and water. Precession angle-frequency curves for (**A**) 0.6%, 0.4%, and 0.2% MC solutions and (**B**) DI water. The black outlined markers on each of the curves indicate the precession angles that correspond with the peak velocities in Fig. 3A.

To characterize average velocity and precession angle in MC solutions, swimming motions under different frequencies and constant field strength of 11 mT before reaching the step-out frequency were recorded. The step-out frequency is the rotational frequency of the magnetic field that defines the threshold at which the magnetic torque is not sufficient to keep the microswimmer’s rotation synchronous with the field’s rotation; this leads to a decrease in swimming velocity. The preparation and characterization of the MC solutions are in the supplementary material. It was previously demonstrated that the field strength did not affect the trend of the velocity-frequency curves^18^. Due to the lower viscosity of water, less torque is needed for actuation; thus, a field strength of 1 mT was used in the experiments in water (Figs. 3B, 4B).

The velocity-frequency curves of the achiral planar microswimmers in MC solutions (0.2%, 0.4%, and 0.6%) and water, as shown in Fig. 3A,B. The highest frequencies presented for each curve are the respective step-out frequency. For 0.4% and 0.6% MC solutions, the peak average velocities appear before step-out; while for water and 0.2% MC solution, the peak average velocities appear at step-out. The values of the peak average velocities and the step-out frequencies are different for each type of fluids, which is expected due to the different viscosities.

The precession angle-frequency curves, as shown in Fig. 4, were obtained from the same experiments from which the velocity-frequency curves were obtained. Precession angle changes nonlinear with increasing frequency and has a direct effect on the velocity of the microswimmer, which is consistent with previous reports^4,5^. In water, the precession angle decreased close to linearly with frequency. In the 0.2% MC solution, the precession angle decreased linearly from 87.522° to 79.895° between 1 and 13 Hz and then decreased with a steadily increasing slope. On the other hand, in the 0.4% and 0.6% MC solution, the precession angle had a decreasing slope. A representative microswimmer swimming in 0.6% with different precession angles is shown in Supplementary video S2.

Previous studies on achiral planar microswimmers verified that linear velocity profiles and nonlinear velocity profiles can be obtained by maintaining a constant precession angle and varying precession angle respectively^5,6^. According to Figs. 3 and 4, the peak average velocities before step-out in different fluids rely on the precession angle. For instance, the peaks velocities of the microswimmers in 0.2%, 0.4%, and 0.6% are located at 18 Hz, 15 Hz, and 8.5 Hz respectively, which corresponds to the respective precession angles of 49.78 ± 2.49°, 35.75 ± 8.84°, and 34.20 ± 3.79°; the point indicating the precession angle that corresponds to the peak velocities is marked on Fig. 4A. This indicates a result where a precession angle close to 45° yields peak average velocity. Indeed, the peak average velocities from each case can form a linear relationship with frequency in Fig. 3A, which signifies that the linear relationship can be upheld when the precession angle does not deviate significantly; this is consistent with previous studies^5^. The velocities peaked at different frequencies because the microswimmers in different fluids have different precession angles at the same frequencies. This indicates that various fluids' viscosities have an indirect effect on velocity through the direct effect on the precession angle^5^. One can also infer that a microswimmer's velocity at a particular frequency should be similar in any fluids in this study as long as the precession angle remains unchanged. Indeed, the 0.2% curve and the 0.6% curve in Fig. 3A intersect at 6.5 Hz, where microswimmers in both fluids have very similar velocities, just as the corresponding curves in Fig. 4 intersect at 6.5 Hz, where microswimmers in both fluids have the same precession angle. Another intersection of the same nature can be observed around 11 Hz.

## Discussion

The microswimmers can normally swim in the water and different MC solutions; however, as the concentration of the MC solution increased to 0.4% and 0.6%, the microswimmers could not swim below the starting frequencies of 10 Hz and 6 Hz respectively because their precession angles were close or equal to 90°, at which point the microswimmers only exhibited tumbling motion that leads to rolling^6^. The precession angles and velocities below the starting frequency are not plotted in Figs. 3 and 4 since these frequencies did not lead to swimming. After the starting frequency, the microswimmers changed from tumbling motion to wobbling motion where their precession angles gradually decrease from 90° to around 85°, at which point the microswimmers started to swim. The relationship between the precession angle and frequency can be explained using the relationship between the magnetic torque ${\mathbf{T}}_{\mathbf{m}}$ and the hydrodynamic torque ${\mathbf{T}}_{\mathbf{r}}$^5^. The torque balance ${\mathbf{T}}_{\mathbf{m}}={\mathbf{T}}_{\mathbf{r}}$ can be expressed using the stokes approximation as

5 $$\left|\mathbf{m}\right|\left|\mathbf{B}\right|\mathrm{sin}\varphi =-\eta \varvec{{\mathcal{G}}}{\varvec{\Omega}}$$

where $\mathbf{m}$ is the magnetic moment vector, $\mathbf{B}$ is the magnetic field vector, $\varphi$ is the phase lag angle between $\mathbf{m}$ and $\mathbf{B}$, $\eta$ is the dynamic viscosity, $\varvec{\mathcal{G}}$ is the rotational resistance tensors, and ${\varvec{\Omega}}$ is the rotational frequency. The rotational resistance tensor $\varvec{{\mathcal{G}}}$ depends on the precession angle of the achiral planar microswimmer. In Newtonian fluids, when ${\varvec{\Omega}}$ increases**,** ${\mathbf{T}}_{\mathbf{r}}$ also increases; thus, breaking the balance between ${\mathbf{T}}_{\mathbf{r}}$ and ${\mathbf{T}}_{\mathbf{m}}$; as a result, the precession angle undergoes a gradual change to adjust $\varvec{{\mathcal{G}}}$ to maintain the balance, as observed in Fig. 4. For non-Newtonian fluids, the dynamic viscosity $\eta$ is a function of shear rate; therefore, the local dynamic viscosity will change with the rotational frequency of the microswimmer. When ${\varvec{\Omega}}$ increases in non-Newtonian fluids with the rheological behavior of shear thinning, $\eta$ decreases; thus, resulting in ${\mathbf{T}}_{\mathbf{r}}$ to change differently manner from that of a Newtonian fluid. However, changing the precession angle resulted in $\varvec{\mathcal{G}}$ being adjusted to increase ${\mathbf{T}}_{\mathbf{r}}$ in a manner that is similar to the case with Newtonian fluid. As the precession angle changes with frequency, the rate of change in velocity also changes, and the velocity profile becomes nonlinear, as seen in Fig. 3. Evidently, the velocity profile for 0.2% MC solution in Fig. 3A is linear from 1 to 12 Hz, which corresponds to the trivial changes in precession angle from 87.522° to 80.428°, and nonlinear from 12 to 18 Hz, which corresponds to significant changes in precession angle from 80.428° to 49.782°. This observation is consistent with previous studies where large changes in precession angle led to nonlinear velocity profiles^5,19^. This leads to the speculation that the change in viscosity due to interaction between the microswimmer and the polymers locally will not have a significant effect on the relationship between the precession angle and frequency. From the perspective of controlling the microswimmer, the parameter adjustments needed to maintain the precession angle to obtain a linear profile in a Newtonian fluid, as seen in previous work^20^, should not differ much from those needed in non-Newtonian fluids.

**Figure 5:**
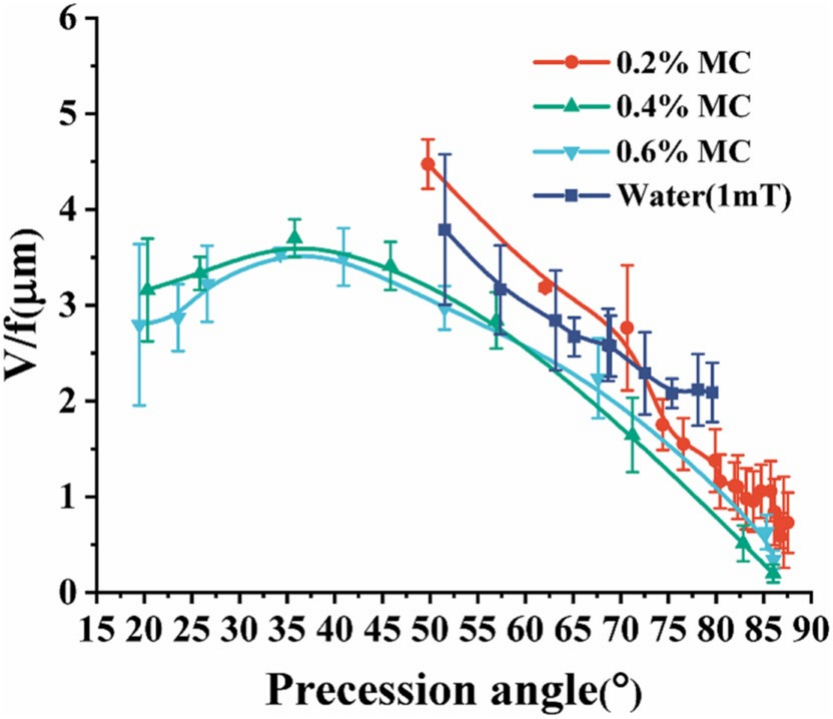
Comparing the dimensionless speeds $V/f$ at different precession angles in different fluidic environments.

The relationship between precession angle, speed, and frequency described from the aforementioned observation can be further explained using a theoretical formula for the velocity of achiral planar microswimmers^13^

6 $$\frac{V}{{fa}} = \widetilde{{Ch}}s_{\psi } s_{{2\theta }}$$

where $V$ is the translational velocity, $f$ is the rotational frequency, *a* is the body length of the achiral planar microswimmers, $\tilde{Ch }$ is a dimensionless chirality matrix, $\psi$ is the Euler angle, and $\theta$ is the precession angle. Here, ${s}_{\psi }$ and ${s}_{2\theta }$ are shorthand notations for *sin*(*ψ*) and *sin*(2*θ*). It can be seen that velocity is proportional to ${s}_{2\theta }$, as validated by Fig. 5. For instance, Eq. (6) states that the precession angles 45° and 90° will yield a maximum velocity and a velocity of 0, respectively. In Fig. 5, the microswimmers across all fluids indeed exhibited maximum and very small dimensionless velocities at precession angles close to 45° and near 90°, respectively.

To further elucidate the relationship between velocity and precession angle, we show the comparison between the dimensionless speeds ($V/af$)^21^ of the microswimmers in different MC solutions. Photolithography-fabricated microswimmers have high structural uniformity; hence, the body length $a$ is constant; thus, the dimensionless speed is simplified to $V/f$. It was observed that the dimensionless speeds with the same precession angle are very similar in three different concentrations of MC solutions and water prior to step-out at precession angles greater than 42.5°, as shown in Fig. 5. As corroborated in Fig. 5, $V/f$ measured in different fluidic environments is indeed reliant on the precession angle and moderately affected by the type of fluids. This, however, is inconsistent with a previous report by Berg et al., which stated that viscous fluids can promote bacteria's motion such as *E. coli*^7^. One of the main reasons for this inconsistency is because bacteria could enter into the porous space of MC and be unhindered by the high viscosity of macro-fluids. On the contrary, the achiral planar microswimmers, with a width of 20 μm, are several times larger than *Escherichia coli* and cannot enter the porous space. Additionally, it can be seen from Fig. 3 that achiral planar microswimmers can still swim at a similar speed after reaching the peak average velocities at 0.4% MC and 0.6% MC, while achiral planar microswimmers step out after reaching the highest speed in the water and 0.2% MC immediately. At the same time, combined with Fig. 4, after achiral planar microswimmers reach the peak average velocities in 0.4% MC and 0.6% MC, the reduction of the precession angle slows down. Finally, the microswimmers maintained steady swimming in 0.4% MC and 0.6% MC when the precession angle is reduced to less than 49.78° while the microswimmers in water and 0.2% MC can only swim when the precession angle is greater than 49.78°, as shown in Fig. 5. A possible reason for this is that achiral planar microswimmers encountered different microenvironments in different fluids; there are more microfibers and particles in 0.4% and 0.6% MC solutions than in 0.2% MC solution and water. The microfiber increased the local resistance torque which made it more difficult to maintain Eq. (5); as a result, the precession angle reduces further in order to reduce resistance and maintain steady rotation. This led to a large range of precession angles when swimming in 0.4% and 0.6% MC solutions.

## Conclusion

In conclusion, the photolithography-fabricated achiral planar microswimmers are promising candidates as microswimmers for biomedical applications because they can be massively fabricated using conventional technologies and have excellent mobility both in bulk fluid and on surfaces. To demonstrate the potential to operate in vivo, the achiral planar microswimmers should operate without being hindered by the non-Newtonian biological fluids. Thus, this work systemically examined the swimming performance of the microswimmers in MC solutions of different concentrations. Experiments with the microswimmers actuated using uniform rotating magnetic fields with constant magnetic strength to characterize their velocities and precession angles under different rotational frequencies. It was demonstrated that the achiral planar microswimmers in Newtonian and non-Newtonian fluids can exhibit similar dimensionless speeds if they have the same precession angle while the local fibrous microenvironment of polymeric fluids might have influence over the range of precession angles for steady swimming. The results demonstrate that while these non-Newtonian fluids have influences on the microswimmers, there are neither enhancement nor significant hindrance to the swimming performance of the photolithography-fabricated achiral planar microswimmers.

## Methods

### MC solution preparation

The preparation method of the MC solutions with w/v concentrations of 0.6%, 0.4%, and 0.2% is described as follows. First, 1.5 g of MC (Sigma Aldrich, M0512) was put into 150 ml of deionized (DI) water. Then, the mixture was thoroughly stirred overnight through magnetic stirring at 1500 rpm at room temperature; this yielded the MC solution. Finally, in order to reduce the amounts of super fibers in the mixture and improve the uniformity of the solution, the MC solution was filtered twice via suction filtration using a filter with a pore size of 10 μm and then a filter with a pore size of 5–13 μm. This yielded a stock MC solution with a mass concentration of 1% w/v. MC solutions with concentrations of 0.6% (w/v), 0.4%, and 0.2% MC solution were prepared by diluting the 1% (w/v) MC solution with DI water. The prepared MC solutions were stored in the refrigerator at 2 ℃.

### Viscosity measurement of MC solution

Using a rotational viscometer (Anton Paar), the shear stress and shear rate relationship of the three methylcellulose solutions were determined, shown in Supplementary Fig S1. A cone in plate geometry, used for low viscous solutions, was used to apply a shear rate to the samples between 1 and 1000 Hz.

### Fabrication and characterization of achiral planar microswimmers

The L-shaped achiral planar microswimmers were prepared through conventional photolithography and lift-off method, as shown in Fig. 2A–C. First, a layer of dextran (10% w/v) and a layer of negative photoresist (SU-8) with 5 μm thickness were spin-coated on a 3-inch silicon wafer in sequence. Then, UV exposure was performed through the mask to transfer the pre-designed patterns onto the photoresist, followed by development in propylene glycol monomethyl ether acetate (PGMEA). Subsequently, electron beam evaporation was used to deposit titanium-cobalt-titanium (thicknesses are 15, 170, and 15 nm, respectively) on the photoresist patterns. The inner titanium layer increased the adhesion between the cobalt layer and photoresist layer; the middle cobalt layer provided magnetism; the outer titanium layers ensured biocompatibility. Finally, the wafer containing achiral-shaped microswimmers was immersed in DI water to dissolve the water-soluble dextran layer and the achiral-shaped microswimmers were not lift-off from the substrate until the dextran layer was dissolved entirely in DI water. An achiral-shaped microswimmer with more details could be observed through an SEM, as shown in Fig. 2D.

### Magnetic actuation, data acquisition, and analysis

A three-dimensional Helmholtz coil system was used to generate uniform rotating magnetic fields to actuate the microswimmers for the experiments; the uniform field exert torque on the microswimmers without introducing translational force. An optical microscope system with a 5 × objective lens and a CMOS camera was mounted on the Helmholtz coil system for the observation and video recording of the microswimmers in motion. The Helmholtz coil system consists of three pairs of electromagnetic coils that are powered by three power supplies (Kepco, BOP20-5M), which were controlled by a Data Acquisition (DAQ) device (National Instruments, PCI-6259). A LabVIEW interface allowed the users to control the DAQ device and the camera. For experiments, the microswimmers was transferred to a PDMS chamber via pipetting and subsequently subjected to uniform rotating magnetic fields generated by the coils for magnetic actuation. The swimming motion of microswimmers were recorded at 100 fps with a resolution of 1280 × 1024 pixels. The system and experimental setup are shown in Supplementary Fig S2. To calculate the swimming speed of microswimmers, the recorded videos were imported into MATLAB where the positions of microswimmers in each frame in the videos were tracked via a tracking algorithm; the positions were then be used to calculate the swimming speed of the microswimmer. To obtain the precession angles of each microswimmer, the frame when the plane is formed by a rotating microswimmer’s easy axis and the rotation axis perpendicular to the focal plane [see Fig. 1B] is analyzed using the angle measurement tool in the software PicPick (NGWIN, v5.1.3).

## Supplementary Information

Supplementary Information.

## Data Availability

The data that support the findings of this study are available from the corresponding author upon reasonable request.
